# Effects of Ions-Releasing Restorative Materials on the Dentine Bonding Longevity of Modern Universal Adhesives after Load-Cycle and Prolonged Artificial Saliva Aging

**DOI:** 10.3390/ma12050722

**Published:** 2019-03-01

**Authors:** Salvatore Sauro, Irina Makeeva, Vicente Faus-Matoses, Federico Foschi, Massimo Giovarruscio, Paula Maciel Pires, Maria Elisa Martins Moura, Aline Almeida Neves, Vicente Faus-Llácer

**Affiliations:** 1 Departamento de Odontologia, Facultad de Ciencias de la Salud, Universidad CEU Cardenal Herrera, 46115 Valencia, SPAIN; 2 Institute of Dentistry, I. M. Sechenov First Moscow State Medical University, 119146 Moscow, Russia; irina_makeeva@inbox.ru; 3 Departamento de Estomatología, Facultad de Medicina y Odontología, Universitat de Valencia, 46010 Valencia, SPAIN; fausvj@uv.es (V.F.-M.); Vicente.J.Faus@uv.es (V.F.-L.); 4 Department of Restorative Dentistry, Faculty of Dentistry, Oral & Craniofacial Sciences at King’s College London, Tower Wing, Guy’s Hospital, Great Maze Pond, London SE1 9RT, UK; federico.foschi@kcl.ac.uk (F.F.); giovarruscio@me.com (M.G.); 5 Department of Pediatric Dentistry, Federal University of Rio de Janeiro, 21941-617 Rio de Janeiro, Brazil; paulinha_pmp@hotmail.com (P.M.P.); aline.neves@odonto.ufrj.br (A.A.N.); 6 Materiais Dentários, Universidade Federal do Ceará, Fortaleza, 60430-355 Ceará, Brazil; mariaelisa_martins@hotmail.com

**Keywords:** adhesion, cycling mechanical stress, dentine, longevity, glass-ionomer cements, universal adhesives

## Abstract

This study aimed at evaluating the microtensile bond strength (MTBS) and fractographic features of dentine-bonded specimens created using universal adhesives applied in etch-and-rinse (ER) or self-etching (SE) mode in combination with modern ion-releasing resin-modified glass-ionomer cement (RMGIC)-based materials after load cycling and artificial saliva aging. Two universal adhesives (FTB: Futurabond M+, VOCO, Germany; SCU: Scotchbond Universal, 3M Oral Care, USA) were used. Composite build-ups were made with conventional nano-filled composite (AURA, SDI, Australia), conventional resin-modified glass ionomer cement (Ionolux VOCO, Germany), or a (RMGIC)-based composite (ACTIVA, Pulpdent, USA). The specimens were divided in three groups and immersed in deionized water for 24 h, load-cycled (350,000 cycles; 3 Hz; 70 N), or load-cycled and cut into matchsticks and finally immersed for 8 months in artificial saliva (AS). The specimens were cut into matchsticks and tested for microtensile bond strength. The results were analyzed statistically using three-way ANOVA and Fisher’s LSD post hoc test (*p* < 0.05). Fractographic analysis was performed through stereomicroscope and FE-SEM. FTB showed no significant drop in bond strength after aging. Unlike the conventional composite, the two RMGIC-based materials caused no bond strength reduction in SCU after load-cycle aging and after prolonged aging (8 months). The SEM fractographic analysis showed severe degradation, especially with composite applied on dentine bonded with SCU in ER mode; such degradation was less evident with the two GIC-based materials. The dentine-bond longevity may be influenced by the composition rather than the mode of application (ER vs. SE) of the universal adhesives. Moreover, the choice of the restorative material may play an important role on the longevity of the finalrestoration. Indeed, bioactive GIC-based materials may contribute to maintain the bonding performance of simplified universal adhesives over time, especially when these bonding systems are applied in ER mode.

## 1. Introduction

Direct restorations in modern operative dentistry are frequently accomplished using conventional resin composites due to their excellent mechanical and aesthetic properties [1,2]. Nevertheless, such restorative materials are still characterized by important downsides associated to polymerization shrinkage; a phenomenon that may induce stress at resin–dentine interfaces during the light-curing procedures and jeopardize their longevity [3,4,5]. Indeed, it has been widely demonstrated that the volumetric contraction of conventional resin composites can transfer polymerization stress directly to the adhesive-bonded interface, causing its innermost deformation due to a lack of proper bonding performance of some adhesive systems [3,6,7]. Consequently, the sealing between composite and dental hard tissues (i.e., dentine and enamel) can be seriously compromised. This will result in gaps and marginal leakage formation, which are pathways for microleakage of oral fluids, bacteria, and enzymes penetration [3,8,9,10]. Such issues may translate into important clinical problems such as post-operative sensitivity, marginal discoloration, recurrent caries, and advanced pulp pathology in all those cases that are seriously compromised by the caries process [11,12].

The recently introduced universal adhesive systems are currently very popular in general dental practices, as well as in dental hospitals, due to the fact that they can be applied both in self-etching (SE) and etch-and-rinse (ER) modes. Considering their compositions, universal adhesives can be also classified as simplified systems because all ingredients, including acidic functional monomers and solvents, are incorporated into one bottle. They are similar to one-step self-etching systems, so that they might still present issues related to bonding performance, degradation, and longevity [9,13]. However, application in self-etching mode minimizes recontamination of the dentine by blood and saliva during etch washing and drying. This makes SE a less technique-sensitive procedure compared to ER application mode. Moreover, SE systems present further benefits such as less post-operative sensitivity due to residual smear plugs, which are usually only partially removed from inside the dentinal tubules because of the mild acidic nature of SE systems. Indeed, the tubules remain occluded and the dentinal fluid movement is less evident compared to that usually experienced with ER systems [9,11]. 

On the other hand, great attention has been given to improve the effectiveness and longevity of resin–dentine bonds through several clinical strategies that may abate stress concentration at the resin–dentine interface during polymerization [14]. For instance, the use of flowable composites or resin-modified glass-ionomer cements (RMGIC) as liners or as dentine substitute materials may represent a suitable method to provide a sort of “stress-absorption” effect at the bonding interface [15,16]. This has been advocated to prevent stress development at the dentine-bonded interface and reduce gap formation, microleakage, and degradation over time [14,17,18]. Although RMGIC are self-adhesive materials, they are also often applied in dentine after etching and adhesive application, especially in those situations where the structure of the dental crown is highly compromised and a lack of mechanical retention is encountered [19,20,21]. 

It is also important to consider that occlusal stress during mastication, swallowing, as well as in cases of parafunctional habits, can affect the integrity of the bonding interface, making such a structure more susceptible to “quicker” degradation in the oral environment [22]. This seems to be of particular interest in modern, minimally invasive therapeutic restorative dentistry since it has been demonstrated that cyclic mechanical stress can promote gap formation at the margins along the composite restorations; bacteria penetration into narrow marginal gaps might ultimately promote secondary caries formation [23]. Recently, it has been advocated that ion-releasing resin-based restorative materials can reduce such biofilm penetration into marginal gaps of simulated tooth restorations; the risk for development and propagation of secondary caries is also reduced [24].

It is widely accepted that glass-ionomer cement (GIC)-based materials have a bioactive ability to release therapeutic ions such as fluoride. The presence of such ions has been associated with long-term caries inhibition when GIC-based materials are applied as a dentine substitute [25,26,27]. Moreover, GIC-based materials are an ideal dentine substitute as their physical properties, such as the coefficient of thermal expansion, dimensional stability, optical properties (i.e., opacity), and microhardness, are very close to that of dentine [28]. ACTIVA BioActive Restorative is a new type of restorative, bioactive, flowable, resin-based composite comparable to RMGICs. It contains fluoro-aluminum silicate particles and polyacid components of glass ionomer that undergo the acid-base setting reaction. Moreover, a bioactive ionic resin matrix is also contained in ACTIVA, which confers both light and chemical polymerization. According to the manufacturer, ACTIVA release calcium, phosphate, and fluoride when in contact with saliva. It has been advocated that restorative materials able to release specific “therapeutic” ions (e.g., calcium, phosphates, fluoride, strontium, and other minerals) into the dental hard tissues may buffer the constant assault of day-to-day ingestion of acidic food and beverages and encourage remineralization along the margins of the restoration with the tooth [29]. However, it is of great relevance that the use of ion-releasing materials in restorative dentistry may contribute to the reduced activity of proteases such as metalloproteinases (MMPs) and cathepsins involved in collagen degradation. Such enzymes are considered one of the main causes for reduction of bonding longevity when simplified bonding systems are applied in dentine with self-etching or etch-and-rinse protocols [30,31]. Moreover, there is a lack of knowledge about the effects of modern ion-releasing materials based on glass ionomer cements on resin–dentine interfaces created using current universal adhesives after mechanical load cycling and prolonged storage in artificial saliva. 

Thus, the aim of this study was to evaluate, after short-term load-cycle aging or after load-cycle stress followed by prolonged aging (8 months) in artificial saliva (AS), the microtensile bond strength (MTBS) of resin–dentine bonded specimens created using universal adhesives applied in an etch-and-rinse or self-etching mode in combination with modern ion-releasing RMGIC-based materials. Fractographic analysis was also performed using field-emission scanning electron microscopy (FE-SEM).

The hypothesis tested was that compared to conventional resin composite, the use of modern ion-releasing materials would preserve the bonding performance of modern universal adhesives, applied in etch-and-rinse or self-etching, after mechanical load cycling and/or prolonged storage in artificial saliva (8 months).

## 2. Materials and Methods

### 2.1. Preparation of Dentine Specimens and Experimental Design

Sound human molars were extracted for periodontal or orthodontic reasons (ethical approval number: LEC № 11.18, 05/12/2018) and stored in distilled water at 5 °C for no longer than 3 months. The roots were removed 1 mm beneath the cemento–enamel junction using a diamond blade (XL 12205; Benetec, London, UK) mounted on a low-speed microtome (Remet evolution, REMET, Bologna, Italy). A second parallel cut was made to remove the occlusal enamel and expose mid-coronal dentine.

Three main groups (n = 72 specimens/group) were created based on the restorative materials used in this study: (i) RC: Resin composite (Aura SDI, Bayswater Victoria, Australia), applied in 2 mm increment layers up to 6 mm, and light-cured as per manufacturer’s instructions; (ii) RMGIC: Resin-modified glass-ionomer cement (Ionolux; VOCO GmbH, Cuxhaven, Germany) mixed for 10 s in a trituration unit and applied in bulk. Two capsules of RMGIC were used and each one was light-cured as per manufacturer’s instructions to obtain 6 mm build-ups; (iii) ACTIVA (ACTIVA BioActive Restorative, PULPDENT, Watertown, MA, USA) applied in 2 mm increment layers up to 6 mm and light-cured as per manufacturer’s instructions. Light-curing was performed using an light-emitting diode (LED) light source ( >1000 mW/cm^2^) (Radii plus, SDI Ltd., Bayswater Victoria, Australia). The experimental design of this study required that the specimens in each main group were subsequently subdivided into four sub-groups (n = 18 specimens/group) based on the protocol employed for bonding procedures. Two modern universal adhesives were employed in this study: SCU (Scotchbond Universal, 3M Oral Care, St. Paul, MN, USA); FTB: (Futurabond M+, VOCO, Cuxhaven, Germany). These adhesives were applied as per manufacturer’s instructions in self-etching (SE) or in etch-and-rinse (ER) mode (Table 1). In groups SCU–ER and FTB–ER, dentine was etched with 37% orthophosphoric acid for 15 s and subsequently rinsed with distilled water (15 s) and blotted, leaving the substrate moist. Adhesives were light-cured for 10 s. In groups SCU–SE and FTB–SE, the adhesives were applied with a microbrush for 20 s and air dried for 5 s to evaporate the solvent. These were finally light-cured for 10 s using am LED light source ( >1000 mW/cm^2^) (Radii plus, SID Ltd., Bayswater VIC, Australia). The specimens were finally restored with the selected restorative materials as aforementioned in the main groups. At this point, the specimens in each sub-group were furtherly divided into three groups (n = 6 specimens/group) based on the aging protocol: CTR: no aging (control, 24 h in deionized water); LC: Load cycling (350,000 cycles in artificial saliva); LC–AS: Load cycling (350,000 cycles in artificial saliva), followed by prolonged water storage (8 months in artificial saliva). A detailed description of the test groups can be found in Table 2 (Experimental design). The composition of the artificial saliva was AS: 0.103 g L^−1^ of CaCl_2_, 0.019 g L^−1^ of MgCl_2_·6H_2_O, 0.544 g L^−1^ of KH_2_PO_4_, 30 g L^−1^ of KCl, and 4.77 g L^−1^ HEPES (acid) buffer, pH 7.4] [32]. The specimens in the subgroup LC and LC–AS were mounted in plastic rings with acrylic resin for load cycle testing. A compressive load was applied to the flat surface (3 Hz; 70 N) using a 5 mm diameter spherical stainless-steel plunger attached to a cyclic-load machine (model S-MMT-250NB; Shimadzu, Tokyo, Japan) while immersed in AS [18,33].

### 2.2. Micro-Tensile Bond Strength and Failure/Fractographic Analysis

The specimens were cut after the aging period using a hard-tissue microtome (Remet evolution, REMET, Bologna, Italy) across the resin–dentine interface, obtaining approximately 15–18 matchstick-shaped specimens from each tooth (Ø 0.9 mm^2^). These were submitted to microtensile bond strength tests using a device with a stroke length of 50 mm, peak force of 500 N, and a displacement resolution of 0.5 mm. Modes of failure were evaluated at 50× magnification using stereoscopic microscopy and conveyed in a percentage of adhesive (A), mixed (M), or cohesive (C) bonding fracture. Five representative fractured specimens from each sub-group were mounted on aluminum stubs with carbon glue after the critical-point drying process. The specimens were gold-sputter-coated and analyzed using field-emission scanning electron microscopy (FE-SEM S-4100; Hitachi, Wokingham, UK) at 10 kV and a working distance of 15 mm.

Bond strength values in MPa were initially assessed for normality distribution and variances homogeneity using Kolmogorov–Smirnov and Levene’s tests, respectively. Data were then analyzed using a three-way Analysis of Variance (ANOVA Factors: restorative material, adhesive, and aging protocol) and Newman–Keuls multiple-comparison test (α = 0.05). SPSS V16 for Windows (SPSS Inc., Chicago, IL, USA) was used.

## 3. Results

### Micro-Tensile Bond Strength (MTBS) and Failure Mode Analysis

There were no pre-test failures before the microtensile bond strength assessment. Three-way ANOVA revealed a significant effect of adhesive (F = 28.75, *p* < 0.001) and restorative material (F = 6.68, *p* < 0.001) on the bond strength, whereas the aging protocol was not statistically significant (F = 8.17; *p* = 0.125). The interactions between the three variables were significant (*p* < 0.001).

The results of the microtensile bond strength test (mean and ± SD) are depicted in Table 3. It was observed that there was no significant difference (*p* > 0.05) at 24 h testing between the two adhesives when applied in etch-and-rinse (ER) or self-etching (SE) mode and then restored using the conventional RC or the two RMGIC-based materials (IONOLUX and ACTIVA). Conversely, the specimens created with the conventional RMGIC presented no significant differences (*p* > 0.05) when bonded using the two adhesives applied in ER or SE mode. However, all the specimens created with the conventional RMGIC showed a significant lower bond strength compared to those created with RC or ACTIVA. The failure mode showed that all the specimens restored with the RMGIC failed mainly in the cohesive mode, leaving a clear presence of the material still bonded to the dentine. The specimens created with RC or ACTIVA failed mainly in the cohesive in composite and mixed mode, leaving part of the dentine still covered by the restorative material and the other part exposed. 

**Figure 1:**
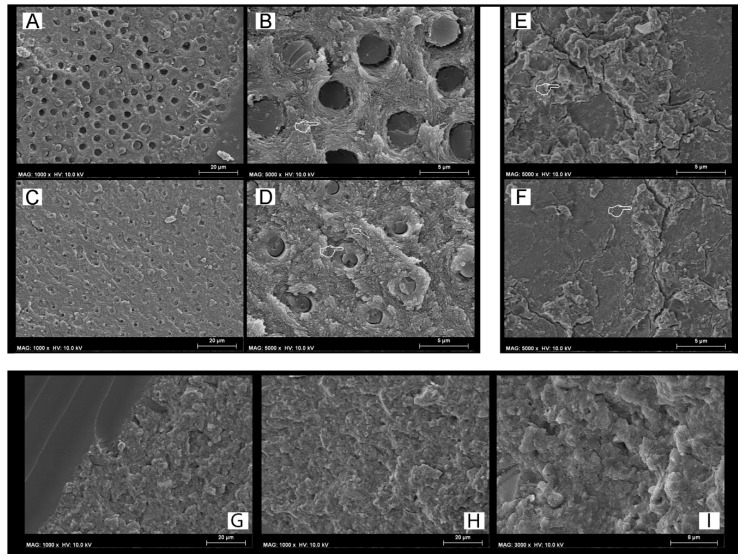
SEM fractographic analysis of the control specimens. (**A**) SEM fractography of a specimen created with SCU applied in ER mode and restored with resin composite (RC) showing the presence of exposed dentine and several resin tags still in the dentinal tubules. (**B**) At higher magnification, it is possible to note the presence of resin tags inside demineralized dentine tubules and collagen fibrils not well infiltrated by the SCU adhesive (pointer). This latter morphological characteristic may indicate that such resin–dentine interface would be affected by degradation over time and would drop in bond strength. (**C**) SEM fractography of a specimen created with FTB applied in ER mode and restored with ACTIVA showing the presence of exposed dentine and several resin tags still inside the small lumen of the dentinal tubules. (**D**) At higher magnification it is possible to observe a typical failure occurred at the bottom of the hybrid layer (HL) characterized by the presence of mineralized peritubular dentine (pointer), with tubules totally obliterated by resin tags and with no presence of demineralized exposed collagen fibrils. Conversely, the dentine specimens bonded with SCU (**E**) and FTB (**F**) applied in ER mode and restored with the RMGIC show the presence of the remaining RMGIC that totally covered the dentine surface. (**G**) SEM fractography of a specimen created with SCU applied in SE mode and restored with ACTIVA and (**H**) FTB applied in SE mode and restored with RC showing a characteristic failure in mixed mode. Note the presence of the remaining resin (**G**) and smear layer on the dentine surface; the latter was even more evident at higher magnification (**I**).

The fractographic analysis showed that the restorative materials employed in this study had no influence on the outcomes in the control storage period (24 h), but all those specimens created with SCU in ER mode presented less resin infiltration within exposed acid-etched dentine collagen fibrils (Figure 1A,B), while the specimens bonded using the FTB applied in ER mode presented fractures mainly underneath the hybrid layer (Figure 1C). Moreover, in this latter case, there was mineralized peri-tubular dentine around the lumen of the dentine tubules and no demineralized and exposed collagen fibrils (Figure 1D). Conversely, all the specimens bonded with the two adhesives applied in SE or ER mode and then restored with RMGIC showed a surface still covered by the restorative material (cohesive mode within RMGIC) with no exposure of the dentine (Figure 1E,F) after microtensile bond strength testing. Furthermore, the fractographic analysis showed that the specimens created both with SCU (Figure 1F) and FTB (Figure 1G) applied in SE, and that failed in mixed or adhesive mode, presented a dentine surface still covered by a smear layer with no presence of collagen fibrils and/or exposed dentinal tubules (Figure 1I).

**Figure 2:**
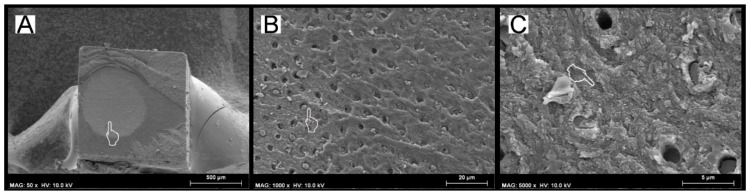
SEM Fractographic analysis after load-cycle aging. (**A**) SEM fractography of a specimen created with SCU applied in ER mode and restored with RC showing a characteristic failure in mixed mode. The finger pointer indicates a brighter area of greater and more evident aging (pointer), which was probably induced by the cycling load. However, when we observed that specific area at higher magnifications (**B**), it was possible to observe that the fracture occurred underneath the hybrid layer (pointer), which is characterized by the presence of mineralized dentine (pointer), with tubules totally obliterated by resin tags and with no presence of demineralized exposed collagen fibrils (**C**).

After submitting the specimens to load-cycle aging, the only group that showed a significant bond strength drop (*p* < 0.05) was that created with the SCU applied in ER mode and restored using the conventional RC. In this group, an important change in the failure mode was also observed; only 15% of the specimens failed in cohesive mode, while failure in mixed and adhesive modes were 55% and 30%, respectively (Table 3). This situation was not evident in the specimens bonded with the same adhesive but restored using IONOLUX (RMGIC) or ACTIVA; no significant bond strength drop (*p* > 0.05) and no radical change in failure mode was observed. The SEM fractography showed no important ultra-morphological changes in most of the fractured resin–dentine interfaces of these groups compared to the control group. Conversely, the specimens created with the SCU applied in ER mode (Figure 2A) and restored with the conventional RC, which failed prevalently in mixed and adhesive mode, showed that the fracture occurred underneath the hybrid layer with no sign of demineralized and/or poorly infiltrated collagen fibrils (Figure 2B,C). 

**Figure 3:**
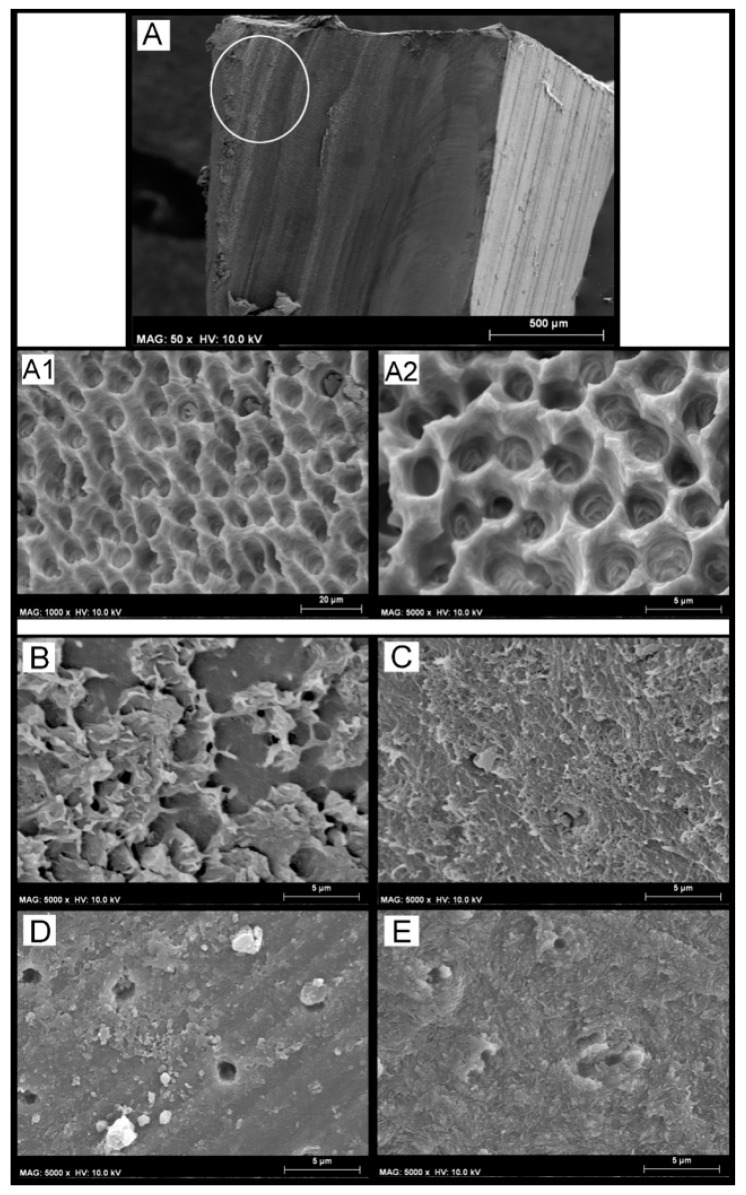
SEM Fractographic analysis after load cycling and aging in artificial saliva. (**A**) SEM fractography of a specimen created with SCU applied in ER mode and restored with RC showing a characteristic failure in adhesive mode. Note that the white circle indicates no physical difference in the material; it was added to show the reader that images (**A1**,**A2**) were obtained by higher magnification in that zone. Indeed, in (**A1**,**A2**) it is possible to see severe collagen degradation without the presence of any resin residual. (**B**) SEM fractography of a specimen created with SCU applied in SE mode where it is possible to see a failure between composite and adhesive, probably due to degradation induced by excessive water sorption upon mechanical stress and prolonged AS storage. However, it was also possible to see, in those specimens that failed in mixed mode, signs of degradation of the collagen fibrils underneath the hybrid/interdiffusion layer (**C**). (**D**) SEM fractography of a specimen created with SCU applied in ER mode and restored with ACTIVA showing that the failure occurred underneath the hybrid layer, but the exposed dentine is well mineralized with no sign of exposed demineralized collagen fibrils. Note also the presence of mineral debrides that are a possible result of the bioactivity of ACTIVA, which released ions and diffused through the resin-bonded dentine. (**E**) SEM fractography of a specimen created with FTB applied in SE mode and restored with RMGIC. The specimens of this group failed mainly in cohesive and mixed mode; this latter zone is characterized by a fracture occurring underneath the hybrid layer, leaving behind a dentine surface completely mineralized with no sign of exposed, denatured, or demineralized collagen fibrils. Please note the presence of a well mineralized intratubular dentine inside the lumen of the dentine tubules.

The prolonged aging in artificial saliva performed subsequent load-cycling stress induced important changes on microtensile bond strength as well as on the ultramorphology of the fracture of some specific groups. In particular, the specimens bonded with SCU applied both in ER and SE mode and then restored with the conventional composite had a significant drop in bond strength compared to the specimens in the groups CTR and LC (*p* < 0.05). Moreover, the number of failures in mixed and adhesive modes increased in the aforementioned groups compared to the control (CTR) group. The SEM fractography showed evident signs of dentine degradation in the group of specimens created with SCU applied in ER mode and then restored with the conventional composite (Figure 3(A1,A2)). The SEM fractography showed that specimens created with SCU applied in SE mode and then restored with the RC presented degradation both of the adhesive (Figure 3B) and dentine hybrid layers (Figure 3C). Conversely, the same specimens restored with the RMGIC or ACTIVA presented a stable bond strength with no significant drop (*p* < 0.05), and the type of failure remained quite similar to the control group. The SEM fractography showed no drastic changes in all those groups for the ultramorphology of fractured resin–dentine interfaces compared to the control group (Figure 3D,E). In particular, the SEM fractography of a specimen created with SCU applied in ER mode and restored with ACTIVA and RMGIC showed the presence of dentine that was well mineralized with no sign of demineralized collagen fibrils, but with the presence of mineral debrides as a possible result of the bioactivity of such GIC-based materials (Figure 3D).

## 4. Discussion

This study showed that the use of modern ion-releasing materials such as conventional RMGIC or RMGIC-based composite (ACTIVA) preserved the bonding performance of only one (SCU) of the two modern universal adhesives bonded to dentine in etch-and-rinse or self-etching mode, after the two aging protocols employed in the experimental design. Conversely, the dentine-bonded specimens created with the FTB universal bonding system applied in etch-and-rinse or self-etching showed no significant drop in bonding performance after aging, regardless the restorative material employed or the aging protocol. Hence, the hypothesis tested in this study needs to be partially accepted as the use of a specific new generation universal bonding systems may confer a stable dentine-bonded interface over time. Nevertheless, the use of modern ion-releasing restorative materials such as RMGIC or ACTIVA may preserve the bonding performance of those universal adhesives that are more prone to degradation after aging.

The effects of the load-cycle aging protocol on the bonding performance of the SCU system applied in ER mode and restored with the conventional RC were relevant; the bond strength of this group of specimens dropped significantly (*p* < 0.05). Moreover, only the specimens bonded using SCU applied both in ER and SE mode and then restored with the conventional composite showed a significant drop in bond strength compared to the specimens in the control (CTR, 24 h) group after prolonged aging in artificial saliva. The ultramorphology analysis performed in the specimens of the control group (24 h), created using the SCU system applied in ER mode and restored with RC showed the presence of demineralized-acid-etched dentine collagen that was not well resin-infiltrated (Figure 1A,B). While the same specimens submitted to LC aging showed no exposed collagen, but mineralized dentine with resin tags that obliterated the dentinal tubules (Figure 2). This was an interesting result, so we hypothesize that a possible explanation to the difference in bonding performance observed between these two latter situations (LC-only aging vs. CTR) may be attributed to the fact that the hybrid layer created using simplified adhesives applied in etch-and-rinse mode can represent the critical part of the resin–dentine interface, as it probably remains only partially polymerized [33,34,35]. Indeed, it has been advocated that during cycling loading such un-polymerized monomers within the hybrid layer, created with simplified, highly hydrophilic etch-and-rinse adhesives, may be mechanically “intruded” into the demineralized dentine causing a more compact and performant hybrid layer. However, such a morphological change within the resin–dentine interface may favor higher stress concentrations during the cycling load at the bottom of the hybrid layer, causing an accelerated mechanically-induced degradation phenomenon in this specific zone that often remains partially demineralized and poorly infiltrated by adhesive monomers [34,35]. Indeed, the absence of a proper, partially demineralized bottom of the hybrid layer may explain why the dentine bonded with SCU or FTB in SE mode showed no bond-strength drop after load-cycle aging, regardless of the restorative material and the protocol employed for aging [33,34]. Our results seem to be in accordance with those of Dorfer et al. [36] who demonstrated water diffusion within the resin–dentine interface and hybrid layer during flexure; this promoted chemical/mechanical degradation and washout of “poorly” polymerized water-soluble monomers.

Apparently, such type of degradation mentioned above was improbable in dentine etched with phosphoric acid and bonded using the same simplified adhesive (SCU), but restored with RMGIC or ACTIVA. Indeed, such restorative materials may have absorbed some of the stress generated by the load-cycle aging due to their lower modulus of elasticity, thereby reducing the risk for degradation at the bonding interface [14,15]. The fact that the two GIC-based materials with lower moduli of elasticity may have distributed stresses within their bulk structure lowering the tension concentration at the interface created with the SCU adhesive, applied both in ER and SE mode and subsequently submitted to a cycling load followed by prolonged storage in AS. This observation was supported by the absence of reduction in bonding performance compared to those specimens restored with the conventional composite; this latter group presented a significant bond strength drop (*p* < 0.05) after such a prolonged aging protocol. In addition to the significant bond strength reduction (Table 3), the results of this current study also showed the presence of funneled dentinal tubules, with no presence of collagen fibrils and no residual of restorative material on the dentine surface (Figure 1), which are all typical morphological signs that indicate collagen hydrolysis and proteolytic denaturation caused by the activity of proteases such as MMPs and cathepsins [34,35,37]. Conversely, the SCU adhesive applied in ER mode and restored with ACTIVA failed mainly in mixed mode or in cohesive/mixed mode when restored with the RMGIC. The SEM fractographic analysis highlighted in those specimens the presence of exposed dentine due to a fracture that occurred underneath the hybrid layer, which left behind a well mineralized dentine with no sign of collagen degradation. Indeed, in this latter case, mineralized peri-tubular dentine around the lumen of the dentine tubules and with no demineralized and exposed collagen fibrils was often observed; this is a typical ultramorphological aspect of failure occurring away from the hybrid layer in resin–dentine interface characterized by high bonding stability [37]. 

Furthermore, mineral debris were detected as a possible result of the bioactivity of ACTIVA and RMGIC (Figure 3D). Indeed, glass-ionomer materials are considered the main bioactive ion-releasing restorative materials currently available in clinics, since they may be able to induce mineral growth within the bonded-dentine interface [18]. We speculate that the results of this study may be somehow correlated to the those hypothesized by Toledano et al. [22,33], who showed that when bioactive materials are submitted to mechanical cycling load, they may promote diffusion of ions through the adhesive-bonded dentine due to the permeable nature of simplified all-in-one bonding systems [37], increasing the mineral–matrix ratio, and reduce nanoleakage and permeability at the resin–dentine interface. Moreover, it has been demonstrated that fluoride ions may inhibit both pro- and active metalloproteinases (MMP-2 and MMP-9) [38], thus reducing the enzymatic degradation at the bonding interface. It may be also possible that in the case of diffusion of calcium and phosphate ions through permeable hybrid layers, these may precipitate and crystallize in complex calcium-phosphates and inhibit MMPs through the formation of a Ca-PO/MMP complex [39].

On the other hand, a possible explanation for the differences in bonding performance attained in this study with the two simplified universal adhesives when restored with a conventional RC may be related to their different chemical compositions. Unlike FTB, the SCU system, which was the only adhesive that both when applied in ER and SE mode in combination with RC presented a significant bond strength drop after prolonged aging protocol, contains a polyalkenoic acid copolymer (PAC). It has been shown that PAC contained in adhesives tends to accumulate primarily on the outer surface of the hybrid layer and creates “isles” between dentine and the adhesive layer [39]. It is also well known that PAC has multiple pendent carboxylic acids along a linear backbone that bind water, which causes important water sorption and solubility. Moreover, the high molecular weight of PAC [40] precludes its penetration into interfibrillar spaces within the acid-etched dentine.

Several reports indicated that simplified adhesives containing relatively high amount of bisphenol A diglycidyl methacrylate (Bis-GMA) in combination with PAC and 2-hydroxyethyl methacrylate (HEMA) do not infiltrate well into acid-etched dentine, so creating HEMA-rich/Bis-GMA-poor hybrid layers. It is also believed that HEMA, mixing with water within the hybrid layer, may produce hydrogels able to absorb water, which in turn enable hydrolytic and enzymatic degradation processes that jeopardize the longevity of resin–dentine interfaces [41,42,43]. Furthermore, it is generally well known that water-containing and acidic, single-bottle, pre-hydrolyzed silane coupling agents have a relatively short shelf life because both water and lower pH media can cause silane to degrade over time [44]. A modern, universal adhesive such as SCU contains both free silane and silaned nanofillers. Thus, we believe that water sorption at the adhesive layer may have accelerated polymer hydrolysis and filler debonding, reducing the durability of its bonding performance [44,45]. 

The information obtained in this study, along with all the observations discussed above, may also be relevant to the contemporary philosophy in atraumatic restorative dentistry. This is based on the preparation of minimally invasive cavities in order to preserve as much sound dental tissue as possible. However, such an ultraconservative intervention should always be followed by restorative treatments performed using therapeutic restorative approaches that protect the resin–dentine interface from degradation processes and prevent the reoccurrence of secondary carious lesions [46,47]. It is well known that the bonding performance of adhesive systems applied to caries-affected dentine (CAD) is not as strong as that attained when such materials are used in sound dentine; the bonding performance seems correlated to the low biomechanical properties of CAD (e.g., modulus of elasticity) [47]. Therefore, such a situation leads to failure of the restoration over time, so that improvements and suitable alternative restorative procedures are necessary in order to improve the durability of the bonding between adhesives and CAD. Wang et al [48] demonstrated distinct differences in the depth of dentine demineralization and degree of adhesive infiltration in non-carious and CAD. Because of the structural alteration and porosities in CAD, deeper, demineralized layers occurred. The deeper the demineralized collagen, the poorer the resin infiltration into the deepest part of the CAD. This resulted in phase separation of resin adhesives and “weak” bond strength. However, Tekçe et al. [49] showed that in such circumstances, the use of flowable resin-based composites, RMGICs, and compomers may provide stronger dentine-bond strength and better margin sealing than conventional glass-ionomer cement and resin composites due to the ability of such materials to dissipate the occlusal stress and the therapeutic effect of ions released over time. 

In conclusion, within the limitations of this study, it is possible to affirm that the choice of appropriate materials from a chemical and mechanical point of view can make a difference on the bonding performance/durability of dentine-bonded interfaces. Indeed, the application of well-formulated modern adhesive systems in combination with ion-releasing dentine-replacement materials might offer to clinicians the possibility to perform more long-lasting adhesive restorations. However, these concepts must be corroborated by future in vivo and/or clinical trial studies in order to evaluate their true suitability in a clinical scenario.

## Figures and Tables

**Table 1:** Adhesive system, composition, and application procedures.

Name	Composition	Application
Scotchbond Universal, 3M Oral Care, USA (lot: 627524)	10-MDP, HEMA, silane, dimethacrylate resins, Vitrebond^™^ copolymer, filler, ethanol, water, initiators, and catalysts (pH 2.7)	1. Apply the adhesive on the surface and rub it for 20 s. 2. Gently air-dry the adhesive for approximately 5 s for the solvent to evaporate. 3. Light cure for 10 s (>500 mW/cm^2^).
FuturaBond M+, VOCO, Germany (lot: 1742551)	HEMA, BIS-GMA, ethanol, Acidic adhesive monomer (10-MDP), UDMA, catalyst ethanol, water, initiators, and catalysts (pH 2.8)	1. Apply the adhesive homogenously to the surface. 2. Rub for 20 s. 3. Dry off the adhesive layer with dry, oil-free air for at least 5 s. 4. Light cure for 10 s (>500 mW/cm^2^).

Abbreviations: 10-MDP 10-methacryloxydecyl dihydrogen phosphate, Bis-GMA bisphenol A diglycidyl methacrylate, HEMA 2-hydroxyethyl methacrylate, UDMA urethane dimethacrylate.

**Table 2:** Experimental design. Distribution of specimens in groups and sub-groups for evaluation via microtensile bond strength (MTBS), interface confocal microscopy, and SEM fractographic analysis. CTR = control, no aging; LC = load-cycling; AS = artificial saliva.

Total Number of Specimens in Main Groups	RESIN COMPOSITE (72 Specimens)			RMGIC (72 Specimens)			ACTIVA (72 Specimens)		
**Number of specimens in sub-groups** **(18/group)**	Number of specimens in aging sub-groups (6/ group)								
SCU–ER: Scotchbond Etch and rinse	CTR 6 spec	LC 6 spec	LC+AS 6 spec	CTR 6 spec	LC 6 spec	LC+AS 6 spec	CTR 6 spec	LC 6 spec	LC+AS 6 spec
FTB–ER Futurabond M+ Etch and rinse	CTR 6 spec	LC 6 spec	LC+AS 6 spec	CTR 6 spec	LC 6 spec	LC+AS 6 spec	CTR 6 spec	LC 6 spec	LC+AS 6 spec
SCU–SE: Scotchbond Self-etch	CTR 6 spec	LC 6 spec	LC+AS 6 spec	CTR 6 spec	LC 6 spec	LC+AS 6 spec	CTR 6 spec	LC 6 spec	LC+AS 6 spec
FTB–SE: Futurabond M+ Self-etch	CTR 6 spec	LC 6 spec	LC+AS 6 spec	CTR 6 spec	LC 6 spec	LC+AS 6 spec	CTR 6 spec	LC 6 spec	LC+AS 6 spec

**Table 3:** The results show the mean (± SD) of the MTBS (MPa) to dentine and the percentage (%) of the failure mode analysis.

	RESIN COMPOSITE			RMGIC			ACTIVA		
	CTR	LC	LC+AS	CTR	LC	LC+AS	CTR	LC	LC+AS
SCU–ER: Scotchbond Etch and rinse	48.9 (7.6) A1 50/45/5	33.5 (5.6) B2 15/55/30	28.1 (5.7) B2 10/50/40	35.1 (7.1) B1 80/20/0	33.4 (7.8) B1 75/20/5	31.1 (8.8) B1 50/35/15	55.3 (6.1) A1 45/55/0	53.1 (7.1) A1 55/40/5	50.1 (6.8) A1 30/50/20
FTB–ER Futurabond M+ Etch and rinse	51.2 (5.9) A1 55/40/5	58.1 (7.3) A1 45/50/5	55.3 (6.5) A1 20/65/15	31.3 (6.7) B1 70/30/0	32.1 (6.6) B1 65/35/0	32.1 (7.1) B1 60/30/5	54.2 (5.7) A1 45/55/0	52.7 (6.2) A1 55/40/5	52.1 (5.6) A1 30/50/20
SCU–SE: Scotchbond Self-etch	45.1 (5.2) A1 45/50/5	44.4 (6.2) A1 40/50/10	34.1 (5.9) B1 10/55/35	32.3 (7.4) B1 70/30/0	34.4 (7.2) B1 65/30/5	29.6 (7.9) B1 50/45/5	46.1 (6.2) A1 40/55/5	49.8 (7.4) A1 30/65/5	49.5 (6.9) A1 45/50/5
FTB–SE: Futurabond M+ Self-etch	49.2 (4.9) A1 40/50/10	48.3 (9.3) A1 45/50/5	45.6 (7.5) A1 25/60/15	34.1 (6.2) B1 75/25/0	31.5 (7.7) B1 70/30/50	30.5 (7.5) B1 60/35/5	48.1 (6.2) A1 40/55/5	51.1 (7.4) A1 45/50/5	50.5 (7.4) A1 45/50/5

Failure mode [Cohesive/Mixed/Adhesive]. The same number indicates no significance in column, while the same letter indicates no significance in row (*p* > 0.05).
